# The use of a vascularized pedicle jejunal graft as a treatment for anastomosis dehiscence after transanal pull‐through, with a combined approach, in two dogs

**DOI:** 10.1002/ccr3.4182

**Published:** 2021-08-21

**Authors:** Ilaria Falerno, Francesco Collivignarelli, Massimo Vignoli, Andrea Paolini, Roberto Tamburro

**Affiliations:** ^1^ Faculty of Veterinary Medicine University of Teramo Teramo Italy

**Keywords:** anastomosis dehiscence, colorectal, jejunal, pull‐through

## Abstract

A pedicled jejunal graft was successfully used to treat a colorectal anastomotic dehiscence, which eliminated the tension on the anastomotic site.

## Graphical abstract

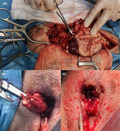

## 1 INTRODUCTION

A novel use of a jejunal graft for the successful repair of a colorectal anastomotic dehiscence after a transanal pull‐through procedure in two dogs.

Intestinal tumors represent 3% of all canine tumors, with 36%‐60% of these affecting the large intestine.^1^, ^2^, ^3^, ^4^ More than 50% of colorectal tumors are malignant, with adenocarcinoma being the most common type.^1^, ^5^, ^6^ Other malignancies of the rectum include leiomyosarcoma, lymphosarcoma, hemangiosarcoma, extramedullary plasmacytoma, mast cell tumor, melanoma, and fibrosarcoma.^3^

Adenocarcinoma can occur in many forms including nodular, pedunculated, or annular constrictive.^1^, ^2^ Single, pedunculated, or polypoid tumors are reported to have a good prognosis after surgical resection, whereas annular colorectal adenocarcinoma is associated with the shortest survival time.^1^, ^7^, ^8^ Malignant tumors require full‐thickness resection with a recommended margin of at least 2‐8 cm. The rectum may be exposed using a ventral, dorsal, lateral, caudal, transanal, transcutaneous, or combined approach. The choice of the approach is made according to the location, size, and type of injury.^1^

A transanal pull‐through procedure is one of the most common surgical approaches used to resect colorectal neoplasms.^6^ A transanal pull‐through or combined abdominal‐transanal approach is well‐described procedures. The postoperative complications after rectal amputation and anastomosis using a transanal pull‐through procedure are tenesmus,^1^, ^9^, ^10^ stricture formation,^1^, ^9^ dehiscence,^6^, ^9^ infection,^9^, ^10^ and fecal incontinence.^1^, ^9^, ^10^ Anastomotic dehiscence usually occurs 3‐5 days after surgery, can be fatal, and requires prompt intervention.^3^ Numerous studies in the human and veterinary literature have focused on the assessment of risk factors, prophylactic measures, and approaches to repair the dehiscence.^11^ A case report was recently published on the use of a temporary end‐on colostomy in a dog as a rescue technique for the repair of an anastomotic dehiscence after a transanal‐rectal pull‐through procedure.^6^ The use of a colostomy has rarely been reported in veterinary medicine,^6^, ^7^ likely because animal owners may not appreciate the associated fecal incontinence and postoperative care requirements.^3^

While the application of jejunal grafts is well‐described in humans,^12^, ^13^, ^14^, ^15^ it is not frequently used in veterinary medicine.^16^, ^17^, ^18^

The aim of this report was to describe the application of a jejunal graft and outcome for the repair of an anastomotic dehiscence after a transanal pull‐through procedure for colorectal carcinoma, in two separate dog cases using a combined approach.

## 2 MATERIALS AND METHODS

### 2.1 Case 1

A 6‐year‐old, 12‐kg, female mixed‐breed dog underwent a transanal‐rectal pull‐through surgery for adenocarcinoma of the colon.

Three days after surgery, the dog was presented to the Veterinary Teaching Hospital of the University of Teramo with a history of weakness and dysorexia. A physical examination showed evidence of tachypnea (44 breaths/min), mucosal pallor, abdominal pain, and hyperthermia (40.3°C). Dehiscence of a previously placed anastomotic suture was suspected. Digital rectal palpation confirmed the presence of a partial ventral‐left‐lateral dehiscence of the colorectal anastomosis localized approximately 1.5 cm from the anus. Laboratory findings showed mild hypoalbuminemia (2.4 g/dL; reference range, 2.8‐3.7 g/dL) and leukocytosis (22 × 10^3^/mm^3^; reference range, 6‐17 × 10^3^/mm^3^). The packed cell volume was 39% (reference range, 35%‐55%), and total protein level was 6.7 g/dL (reference range, 6‐7.5 g/dL). Lateral and dorsoventral abdominal radiographs showed decreased serosal surface visualization with a ground‐glass appearance in the caudal abdomen. The ultrasound examination showed a moderate amount of free peritoneal fluid with hyperechoic fat in the caudal abdomen. Fluid samples were collected by ultrasound‐guided abdominocentesis. Degenerate neutrophils and vegetable material were found among the cytological findings.

The dog was hospitalized for surgical revision. During the hospitalization, the dog received maintenance fluid therapy with lactated Ringer's solution (4 mL/kg/h), methadone (0.2 mg/kg, every 4 hours, administered according to the Glasgow pain scale), and metronidazole‐spiramycin (10 mg/kg, every 12 hours, administered orally).

### 2.2 Case 2

A 5‐year‐old, 33‐kg, female golden retriever was referred to the Veterinary Teaching Hospital for exercise intolerance, dyschezia, tenesmus, and hematochezia. A colorectal surgery was performed 6 days earlier using a transanal pull‐through procedure for colorectal carcinoma. A physical examination showed evidence of severe abdominal pain, mucosal pallor, tachycardia (150 beats/min), and a rectal temperature of 39.7°C. Dehiscence of a previous colon surgery with anastomosis was suspected. Digital intraluminal rectal palpation confirmed the presence of a partial ventral dehiscence of the colorectal anastomosis localized approximately 1 cm from the anus. Blood tests were normal. An abdominal radiographic study in two orthogonal views and abdominal ultrasonography findings were compatible with moderate peritoneal effusion. After abdominocentesis, a cytological evaluation of the fluid showed degenerate neutrophils and intracellular cocci.

The dog was hospitalized for surgical revision. During the hospitalization, the dog received maintenance fluid therapy with lactated Ringer's solution (4 mL/kg/h), methadone (0.2 mg/kg, every 4 hours, administered according to the Glasgow pain scale), and metronidazole‐spiramycin (10 mg/kg, every 12 hours, administered orally).

### 2.3 Surgical procedure

Each dog was slowly administered dexmedetomidine (1 µg/kg) and methadone (0.2 mg/kg) intravenously. Anesthesia was induced intravenously with propofol (initial bolus of 2 mg/kg over 60 seconds followed by incremental bolus doses of 0.5 mg/kg) and maintained using isoflurane in oxygen. A urinary catheter was placed, and the metacarpal artery was catheterized. The intraoperative blood pressure was monitored using both invasive and noninvasive techniques. Cefazolin (22 mg/kg) was administered intraoperatively at induction and every 90 minutes thereafter. A loading dose of meloxicam (0.2 mg/kg) was administered intravenously following induction. Intraoperatively, the dogs received a loading dose of lidocaine (2 mg/kg) intravenously followed by lidocaine CRI (33 µg/kg/min) and fluid therapy with lactated Ringer's solution (6‐7 mL/kg/h).

Each dog was positioned in dorsal recumbency, and both the ventral abdomen and the perineal area were prepared for surgery. A ventral midline abdominal approach was used, starting from the xiphoid to the pubis, and showed a moderate amount of peritoneal effusion.

**FIGURE 1:**
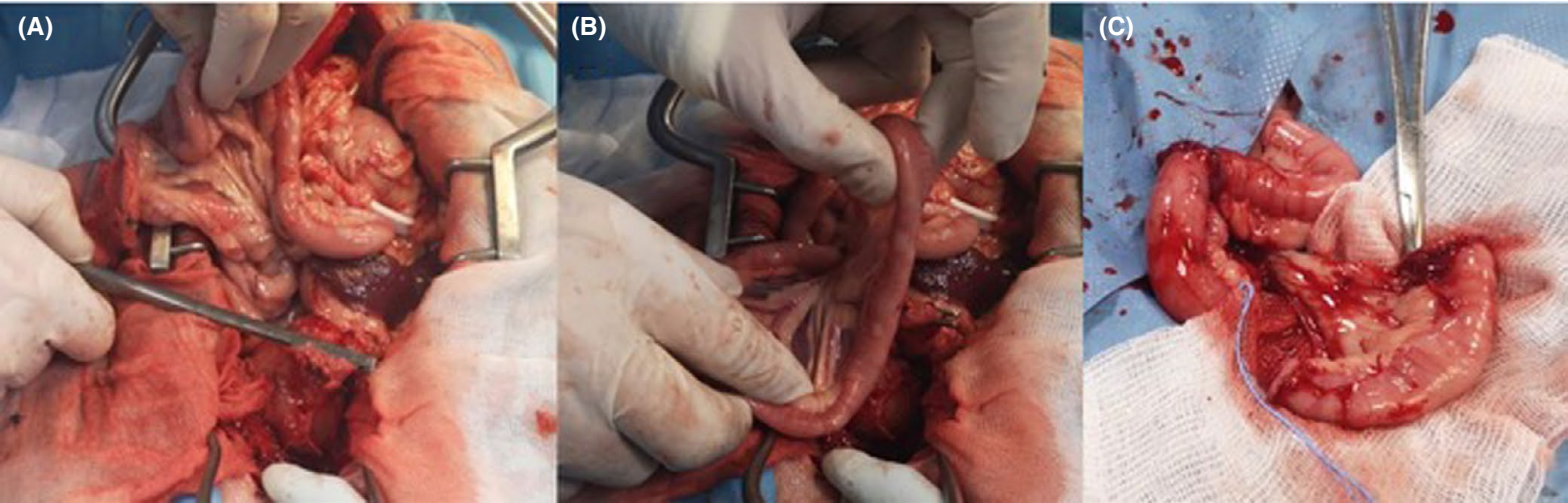
Celiotomy in a 6‐year‐old, 12‐kg, female, mixed‐breed dog. Anastomotic dehiscence of the previous colorectal site (A). Exteriorization and choice of a healthy 5‐cm jejunal segment to be grafted (B). Isolation of the jejunal segment, including the jejunal artery and vein, and end‐to‐end anastomosis of the graft donor site with a 4‐0 polydioxanone suture using a simple interrupted pattern

**FIGURE 2:**
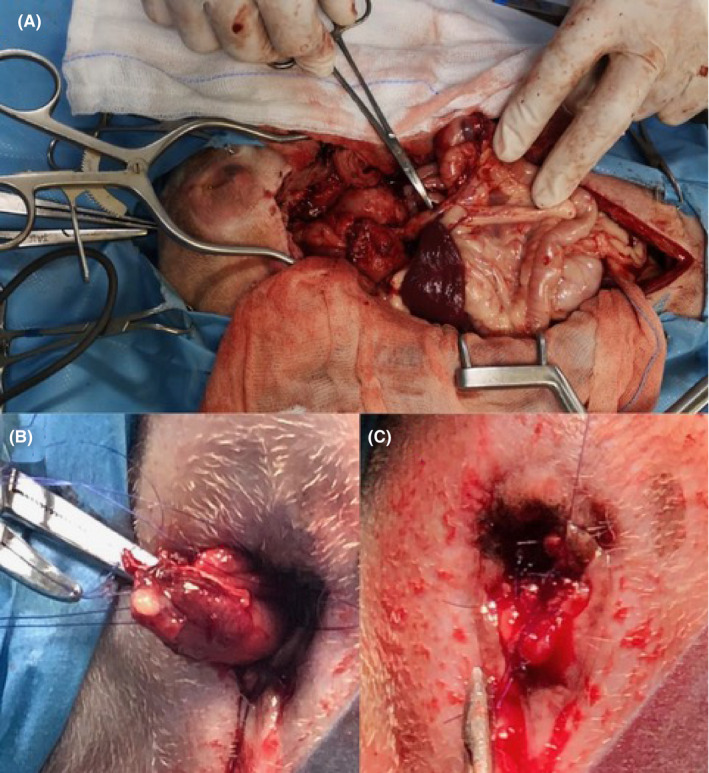
Combined transanal‐abdominal surgical procedure in a 6‐year‐old, 12‐kg female mixed‐breed dog. A Doyenne clamp was inserted through the anus into the abdomen by a second surgeon to grasp the closed, free end of the jejunal graft (A) and pull it out through the anus (B). A transanal‐rectal‐jejunal end‐to‐end anastomosis using a 4‐0 polydioxanone suture using a simple continuous appositional pattern and replacement (C)

To increase the colorectal exposure, bilateral pubic and ischial osteotomies were carried out. The division between the right and left adductor muscles was incised sharply, taking care to stay exactly on the midline to minimize the risk of hemorrhage. The adductor muscles were then elevated subperiosteally from the pubis and ischium with a 0.25‐inch Key periosteal elevator. Then, elevation was continued until the obturator nerves and approximately two‐thirds of the obturator foramina were visible. The prepubic tendon was incised along the pubis to the level of the proposed pubic osteotomy sites, and a 2‐mm pin chuck was used to drill two holes on either side of the four proposed osteotomy sites. The osteotomies of the right and left pubis and ischium were then created using a sagittal oscillating saw. The internal obturator nerves were protected with a malleable retractor while the osteotomies were performed. The internal obturator muscle was elevated subperiosteally from the right pubis and ischium, allowing for the resection of the central bony plate to the left. Upon increased colorectal exposure, an anastomotic dehiscence was evident (Figure 1A). Initially, the dehiscence was isolated by sterile gauze. Debridement and primary closure of the colon and rectal stumps were attempted, which resulted in tension upon closure. To decrease the tension on the anastomosis, a jejunal grafting technique was utilized. A 5‐cm segment of healthy jejunum (Figure 1B), including the jejunal artery and vein, was isolated and mobilized from the middle of the jejunum. The graft donor site was closed using an end‐to‐end anastomosis with a 4‐0 polydioxanone suture (PDS) in a simple, continuous appositional pattern (Figure 1C). The pedicled jejunal segment was transposed to the anastomotic site. Cranially, the rectal and jejunal stumps were sutured in place using an end‐to‐end anastomosis with a 4‐0 PDS in a simple interrupted pattern (Figure 2A). The rectal portion was hard to scarify and anastomose using this approach. The use of a transanal technique was thus necessary. The jejunal caudal stump was closed with a 4‐0 PDS using a continuous Parker‐Kerr inverting suture technique. A Doyenne clamp was inserted through the anus into the abdomen by a second surgeon to pull out the caudal jejunal stump through the anus (Figure 2B). The abdomen was lavaged with 5 L sterile saline, and a Jackson Pratt drain was placed in the peritoneal cavity and was closed without complications. The dog was then positioned in sternal recumbency, and the perineal surgical field was prepared using an external operator. The jejunal caudal stump was externalized using a Doyenne clamp, and the Parker‐Kerr suture was removed. A transanal‐rectal‐jejunal end‐to‐end anastomosis was performed using a 4‐0 PDS in a simple continuous appositional pattern. The jejunal‐rectal anastomosis was then replaced through the anus (Figure 2C).

Major complications were classified as a dehiscence of one or both of the anastomotic sites and severe rectal bleeding, whereas minor complications were classified as tenesmus, leakage, diarrhea, and fecal incontinence.

### 2.4 Postoperative management

Postoperatively, both dogs were hospitalized in the intensive care unit. They were maintained on lidocaine CRI (33 µg/kg/min) for 24 hours and received maintenance fluid therapy with lactated Ringer's solution (3‐4 mL/kg/h), metronidazole‐spiramycin (10 mg/kg, every 12 hours, administered intravenously for 5 days), cefazolin (22 mg/kg, every 12 hours, administered intravenously for 8 days), meloxicam (0.1 mg/ kg, every 24 hours, administered intravenously for 4 days), and methadone (0.15 mg/kg, every 4 hours, administered intravenously, according to the Glasgow pain scale). Pain scoring was performed every 2 hours and the dogs resumed oral feeding the day after surgery with four small meals per day of gastrointestinal canned food.

The mean urinary output was 2.5 mL/kg/h in Dog 1 and 3 mL/kg/h in Dog 2, with the urinary output continuously monitored for 4 days after surgery. Postoperative serial ultrasound examinations were performed to monitor for abdominal effusion and peritoneal reaction.

## 3 RESULTS

Self‐limiting diarrhea was observed for 3 days following surgery in both dogs.

The drains were removed, from both dogs, 4 days after the surgery following a decreased peritoneal effusion. By 10 days after surgery, the wound sutures were removed.

The dogs were discharged 5 days after surgery with normal vital parameters and organic function and with an absence of peritoneal effusion. Wet food was recommended for the following two weeks. A follow‐up examination, including complete blood count, serum biochemistry profile, rectal palpation, and abdominal ultrasound, was performed 20 and 40 days after surgery and was followed by interviews with the owners 1 year after surgery. No complications were highlighted at any follow‐up. A physical examination showed normal vital parameters, absence of abdominal pain, and no signs of wall discontinuity by rectal palpation. The absence of peritoneal effusion was detected by an ultrasound exam. During the telephone interviews, the owners were each asked about the general status, appetite, frequency of defecation, and episodes of incontinence of their dog. Notably, a total recovery was reported by the owners.

The surgical time was 120 min for case 1 and 145 min for case 2.

In both dogs, the intraoperative mean arterial pressure never fell below 65 mm Hg, and no major postoperative complications were observed in either surgery. Self‐limiting diarrhea was observed for 3 days after surgery; however, the surgical procedure was successful and allowed for the resolution of clinical signs.

## 4 DISCUSSION

This report describes the use of a vascularized pedicled jejunal graft as a treatment for anastomotic dehiscence after a transanal pull‐through procedure using a combined approach.

Complications following rectal pull‐through procedures are tenesmus,^1^, ^9^, ^10^ rectal bleeding,^1^, ^9^, ^10^ rectal stricture,^1^, ^9^ suture dehiscence,^6^, ^9^ and transient or permanent fecal incontinence.^1^, ^9^, ^10^ In the literature, the incidence rates reported for each of these complications are highly variable. In a study of 74 cases, 78% of patients undergoing a transanal pull‐through procedure developed at least one postoperative complication; fecal incontinence was the most common complication recorded (57%), followed by diarrhea (43.2%), tenesmus (31.1%), stricture formation (21.6%), rectal bleeding (10.8%), constipation (9.5%), dehiscence (8.1%), and infection (5.4%).^9^ Of the six dogs with anastomotic dehiscence, one developed septic peritonitis, three developed pararectal abscesses, and two had mild incisional dehiscence that healed after a second procedure. The dog with septic peritonitis was euthanized, and the cases of pararectal abscesses were resolved with surgical and medical management.^9^ The incidence of dehiscence increases with resections greater than 6 cm,^1^, ^6^, ^19^ usually occurring 3 to 5 days after surgery during the lag phase of the healing process.^3^

The current treatment for suture dehiscence after a transanal‐rectal pull‐through procedure is a temporary end‐on colostomy with meticulous daily stoma management and 60 days of hospitalization.^6^ Initial management using a human fecal collection bag was ineffective; after 90 days, a second surgery was performed to resect the stoma and recanalize the intestine with a colonic end‐to‐end anastomosis. Dermatitis and stoma management were the major complications described in the aforementioned study. Although the procedure was successful, it required extensive postoperative management by the dog's owner.^6^

In humans, several types of jejunal and ileal grafts (pedicled, augmented, free segment, or a combination of these) have been used for the reconstruction of large anatomical defects.^16^ The most common jejunal grafting procedures involve the reconstruction of pharyngolaryngectomy defects.^14^ Additionally, tracheal, duodenal, esophageal,^12^, ^15^ and urethral replacements have been performed with acceptable results and risks of complications related to the grafting sites.^13^

In small animals, reports of jejunal grafting are less frequent. Brourman et al described a successful repositioning of an obstructed ureter using an ileal graft in a cat. Additionally, ileal grafting has proven to be an effective rescue technique for the treatment of severe ureteral impairments where a primary repair is not possible or has failed.^20^

Moreover, Massie et al reported the innovative use of a vascularized pedicled jejunal graft for the successful repair of a large circumferential duodenal perforation in a dog^16^ and Bensignor et al subsequently described this technique in detail.^17^

Previously, Ziaian et al showed how the jejunal pedicled flap and jejunal serosal patch techniques were effective in closing large duodenal defects in dogs; they obtained better histological results using the jejunal pedicled flap than using the jejunal serosal patch.^18^

In these studies, no complications related to the use of the grafts were found. Possible described complications related to the use of jejunal grafts include the formation of stenosis and vascular thrombosis; dysmotility problems have also been reported.^16^

None of these complications were clinically or ultrasonographically detected in our patients. Further, no tension or obstruction of the vascular pedicle was detected intraoperatively due to the great mobility of the mesentery. No postoperative complications such as tenesmus, leakage, dehiscence, and fecal incontinence were observed.

The risk factors for anastomotic dehiscence and leakage include septic peritonitis, hypoalbuminemia, and intraoperative hypotension.^21^

Preoperatively, both dogs showed evidence of peritoneal effusion with degenerative neutrophils and bacteria upon cytological evaluation for anastomotic dehiscence of a previous colorectal surgery. Intraoperatively, the dehiscence was corrected by eliminating the suture under tension, washing the abdomen with 5 L sterile saline, and inserting a Jackson Pratt drain prior of closure. A broad‐spectrum antibiotic therapy was also administered.

Diarrhea is a common finding associated with intestinal tumors^2^, ^5^, ^8^ and represents a possible postoperative complication after the transanal pull‐through procedure.^9^ In both cases, only diarrhea was observed for 3 days following surgery. Furthermore, postsurgical diarrhea is significantly associated with the length of the resected rectal segment compared to the body weight.^9^ Other possible explanations are a decrease in the rectal area available for the absorption of water from the stool before elimination and the establishment of a condition similar to short bowel syndrome or hypermotility syndrome, similar to that observed in humans, which can cause transient postoperative diarrhea.^9^

Therefore, the use of the pedicled jejunal graft allowed for the resolution of the colorectal dehiscence without major complications and eliminated the tension that the anastomotic site was previously subjected to.

To date, colostomy^6^, ^7^ is the only surgical technique described as an alternative to euthanasia for the treatment of colorectal anastomotic dehiscence after a transanal pull‐through procedure. Recently, Cinti et al reported a case of a temporary end‐on colostomy as a rescue technique to repair an anastomotic dehiscence after a transanal pull‐through procedure in a dog^6^; the procedure allowed for fecal diversion, with the aim of reducing tension on the anastomotic site and allowing for the healing of the distal colorectal area. In that study, dermatitis and stoma management were the major complications described.^6^ Other complications reported in the literature include prolapse of the stoma and skin irritation.^7^ Although the procedure was successful, it required extensive postoperative management by the dog's owner.

In our study, we used a pedicled jejunal graft to successfully treat a colorectal anastomotic dehiscence, which eliminated the tension on the anastomotic site. Moreover, the entire procedure is a single surgical solution that did not require extensive postoperative care by the owners. Therefore, the pedicled jejunal graft technique represents a novel and effective alternative for the repair of colorectal anastomotic dehiscence after a transanal pull‐through procedure. Larger studies are needed to investigate the effectiveness and risks of complications associated with this novel surgical procedure.

## 5 ETHICAL APPROVAL

No ethical approval has been required for this study as it was not experimental. A written informed consent has been taken beforehand form the owners.

## CONFLICT OF INTEREST

The authors declare no conflict of interests related to this report.

## AUTHOR CONTRIBUTIONS

Ilaria Falerno: provided clinical care and wrote the case report. Francesco Collivignarelli: performed the surgical treatment, edited the case report, and served as a corresponding author. Massimo Vignoli: revised and contributed to the manuscript. Andrea Paolini performed reference and reviewed the literature search. Roberto Tamburro: edited the case report and approved the final revision of the manuscript.

## Data Availability

No supplementary data are needed for this case report, and no datasets were generated or analyzed during the current study.
